# Chronic exposure to high fat diet triggers myelin disruption and interleukin-33 upregulation in hypothalamus

**DOI:** 10.1186/s12868-019-0516-6

**Published:** 2019-07-10

**Authors:** Hui-Ting Huang, Sheng-Feng Tsai, Hung-Tsung Wu, Hsin-Ying Huang, Han-Hsueh Hsieh, Yu-Ming Kuo, Po-See Chen, Chung-Shi Yang, Shun-Fen Tzeng

**Affiliations:** 1 Department of Life Sciences, College of Bioscience and Biotechnology, National Cheng Kung University, Tainan, Taiwan; 2 Department of Cell Biology and Anatomy, Institute of Basic Medical Sciences, College of Medicine, National Cheng Kung University, Tainan, Taiwan; 3 Department of Family Medicine, National Cheng Kung University Hospital, College of Medicine, National Cheng Kung University, Tainan, Taiwan; 4 Institute of Biomedical Engineering and Nanomedicine, National Health Research Institutes, Zhunan, Miaoli Taiwan; 5 Department of Psychiatry, National Cheng Kung University Hospital, College of Medicine, National Cheng Kung University, Tainan, Taiwan

**Keywords:** Oligodendrocytes, Myelin, IL-33, Glia, Microglia, Obesity

## Abstract

**Background:**

Hypothalamic inflammation including astrogliosis and microglia activation occurs after intake of high fat diet (HFD) in rodent models or in obese individuals. However, the effect of chronic HFD feeding on oligodendrocytes (OLGs), a myelin-producing glial population in the central nervous system (CNS), remains unclear. In this study, we used 8-week old male C57BL/6 mice fed by HFD for 3–6 months to induce chronic obesity.

**Results:**

The transmission electron microscopy imaging analysis showed that the integrity of hypothalamic myelin was disrupted after HFD feeding for 4 and 6 months. Moreover, the accumulation of Iba1^+^-microglia with an amoeboid hypertrophic form was continually observed in arcuate nucleus of HFD-fed mice during the entire feeding time period. Interleukin-33 (IL-33), a tissue alarmin upon injury to the CNS, was detected with an increased level in hypothalamus after HFD feeding for 3 and 4 months. Furthermore, the in vitro study indicated that exposure of mature OLGs to IL-33 impaired OLG cell structure along with a decline in the expression of myelin basic protein.

**Conclusions:**

Altogether, our findings demonstrate that chronic HFD feeding triggers hypothalamic myelin disruption in accompany with IL-33 upregulation and prolonged microglial activation in hypothalamus. Given that the addition of exogenous IL-33 was harmful for the maturation of OLGs, an increase in IL-33 by chronic HFD feeding might contribute to the induction of hypothalamic myelin disruption.

**Electronic supplementary material:**

The online version of this article (10.1186/s12868-019-0516-6) contains supplementary material, which is available to authorized users.

## Background

Obesity caused by an excessive dietary intake and insufficient energy expenditure has emerged as a major critical factor for developing cardiovascular disease and metabolic syndrome. The obesity-induced damage to neurons in the central nervous system (CNS) and to the integrity of blood-brain barrier (BBB) increases a risk to induce stroke and Alzheimer’s disease [1, 2]. Given that hypothalamus is responsible for the regulation of food intake and energy expenditure through leptin and insulin action [3], maintenance in homeostasis of hypothalamic neuronal and glial functions is important to prevent the development of obesity-associated diseases.

A low-grade inflammation in the peripheral tissues of obese individuals have been well addressed [4–7]. Hypothalamic inflammation also occurs in genetic modified animal models of obesity or high fat diet (HFD)-fed animals [5, 8–10]. It has been reported that an increase in the proinflammatory cytokines and chemokines (i.e. IL-1β, TNF-α, and IL-6) was observed in hypothalamus at the acute (hours) and subacute (weeks) time points after HFD feeding [11, 12]. The activation of microglia and astrocytes, referred as gliosis, has been known to play a critical role in the regulation of HFD-induced resistance to leptin and insulin in company with the disturbance of energy homeostasis [11–14]. The loss of oligodendrocytes (OLGs), a myelin-producing glial population in the central nervous system (CNS), has been detected in the spinal cord at 7 week after HFD feeding [15]. Yet, little is known about the response of hypothalamic OLGs to chronic HFD feeding.

The aim of this study was to examine the response of hypothalamic OLGs to chronic HFD feeding using our established animal model of metabolic disorders by feeding HFD to induce insulin resistance and disrupted lipid metabolism [16]. The study was first to show the disruption of myelin microstructure in posterior lateral hypothalamic area at the later time points of HFD feeding. Alternatively, interleukin-33 (IL-33) acts as a cellular alarmin [17], and has been reported that IL-33 is a critical factor for MS development via inhibiting CNS myelination [18]. Interestingly, we found that IL-33 was increased in hypothalamic OLGs and astrocytes after chronic HFD feeding. In conjunction with our in vitro study that exposure of mature OLGs to IL-33 induced the injury of OLG morphology, the findings suggest that impaired myelin microstructure in hypothalamus after HFD feeding might be caused in part by the action of IL-33 molecules increased by chronic HFD feeding.

## Results

### Chronic HFD feeding-induced change in hypothalamic myelin microstructure

**Fig. 1:**
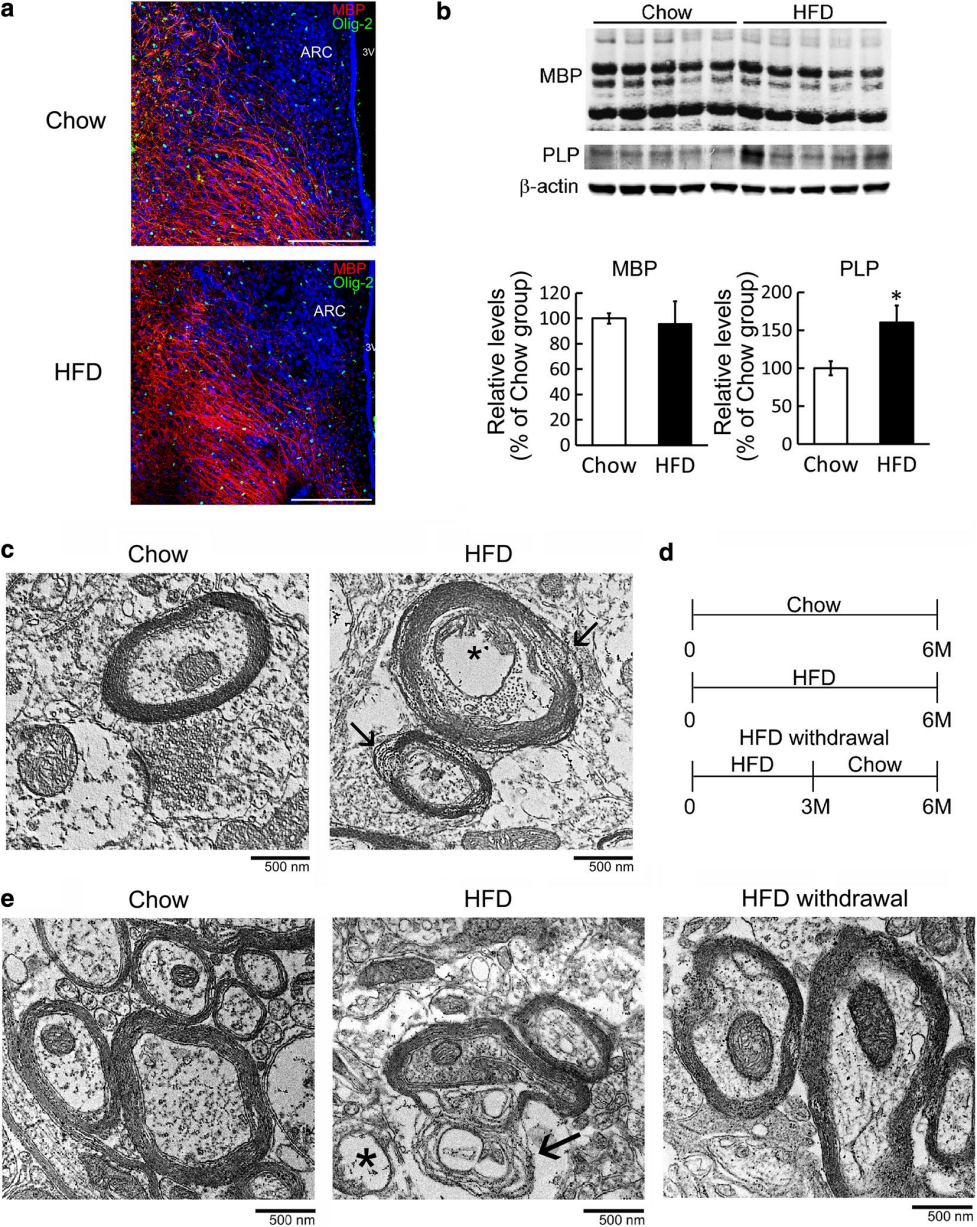
Disruption of hypothalamic myelin structure induced by chronic HFD feeding. **a** Brain tissue sections collected from mice receiving Chow and HFD feeding for 4 months, and then subjected to immunofluorescence for MBP (red) and Olig-2 (green). DAPI nuclear counterstaining (blue) was also conducted. **b** Total proteins were prepared from the hypothalamic tissues collected from animals receiving Chow or HFD for 4 months, and then subjected to western blot analysis for the measurement of MBP, PLP, and β-actin (loading control). The quantification of hypothalamic MBP and PLP level normalized by relative β-actin level was performed. The results are presented as mean ± SEM (n = 5 animals for each time point). *p < 0.05 versus the Chow group. **c** The hypothalamic tissue was removed from Chow and HFD-fed animals at 4 month after feeding (n = 3 animals for each group), and then subjected to TEM imaging analysis. TEM images from the HFD group show the disrupted myelin surrounding the axons (arrows) and swollen/fragmented mitochondria (starlet). **d** The diagram illustrates that mice were fed by Chow and HFD for 6 months, as well as HFD for 3 months and Chow for another 3 months (HFD withdrawal). **e** The hypothalamic tissue sections were prepared from two Chow-fed mice, two HFD-fed mice, and two HFD withdrawal mice for TEM imaging analysis. The representative images indicate that the disorganized myelin was found in the hypothalamic tissue from the HFD-fed group (arrows), and mitochondrial fragmentation was observed (starlet). Yet, hypothalamic myelin structure in the HFD withdrawal group displayed more compact than that observed in the HFD group. Scale bar in **a** 200 μm; in **c**, **e** 500 nm

Loss of myelin-enriched white matter integrity in individuals with a high body mass index (BMI) has been reported through the assessment of diffusion tensor imaging [19]. However, poor information shows if this phenomena takes place in hypothalamus of individuals receiving chronic HFD feeding. Through immunofluorescence to identify OLG-specific transcription factor Olig2 and MBP, we noticed that HFD feeding caused no change in the intensity of MBP immunoreactivity and in the amount of Olig2^+^-OLGs in the hypothalamic region around ARC (Fig. 1a; Additional file 1: Fig. S1). The levels of MBP protein isoforms (14–21.5 kDa) in hypothalamus were not altered by HFD feeding, although myelin proteolipid protein (PLP) levels in hypothalamus were moderately increased after HFD feeding for 4 months when compared to that measured in Chow-fed mice (Fig. 1b). Interestingly, through TEM imaging analysis, the microstructure of myelin in the hypothalamic region (Additional file 1: Fig. S1) was disrupted in HFD-fed group at 4 month post feeding (Fig. 1c, arrows), although a change in the g-ratio between the Chow-fed and HFD-fed groups was insignificant (data not shown). Among myelinated axons shown in TEM images, there was 40% of axons with a disrupted myelin structure in HFD-fed mice, whereas only 10% of axons with myelin disruption was observed in Chow-fed mice. Note that the most of myelin sheath in hypothalamic tissues was compact in the Chow groups (Fig. 1c). These results reveal that HFD feeding for 4 months resulted in the disruption of myelin, but did not yet induce hypomyelination on hypothalamic axons.

Yet, the disrupted myelin sheath was continuously detected in hypothalamus at 6 month post feeding (Fig. 1e, arrows). Since the integrity of myelin structure in the hypothalamic region was less affected by HFD feeding at 3 month (Additional file 1: Fig. S2), we were next to examine if HFD withdrawal at this time point can prevent the disruption of myelin structure in hypothalamus. Thus, we conducted the experiments that mice in the HFD withdrawal group were fed by HFD for the first 3 months and then by Chow for another 3 months (Fig. 1d). Their body weight declined significantly after their diet was changed to Chow (Additional file 1: Fig. S3A). Moreover, their water intake and food intake were back to the level of the Chow group much later (Additional file 1: Fig. S3B and C). In addition, myelin structure in the hypothalamic tissue derived from the HFD withdrawal group was as compact as that observed in the Chow group (Fig. 1e). These results reveal that chronic HFD feeding can damage hypothalamic myelin microstructure, and this disruption can be prevented by changing HFD to the normal diet at the earlier time point.

### Microglia activation in hypothalamic ARC by chronic HFD feeding

**Fig. 2:**
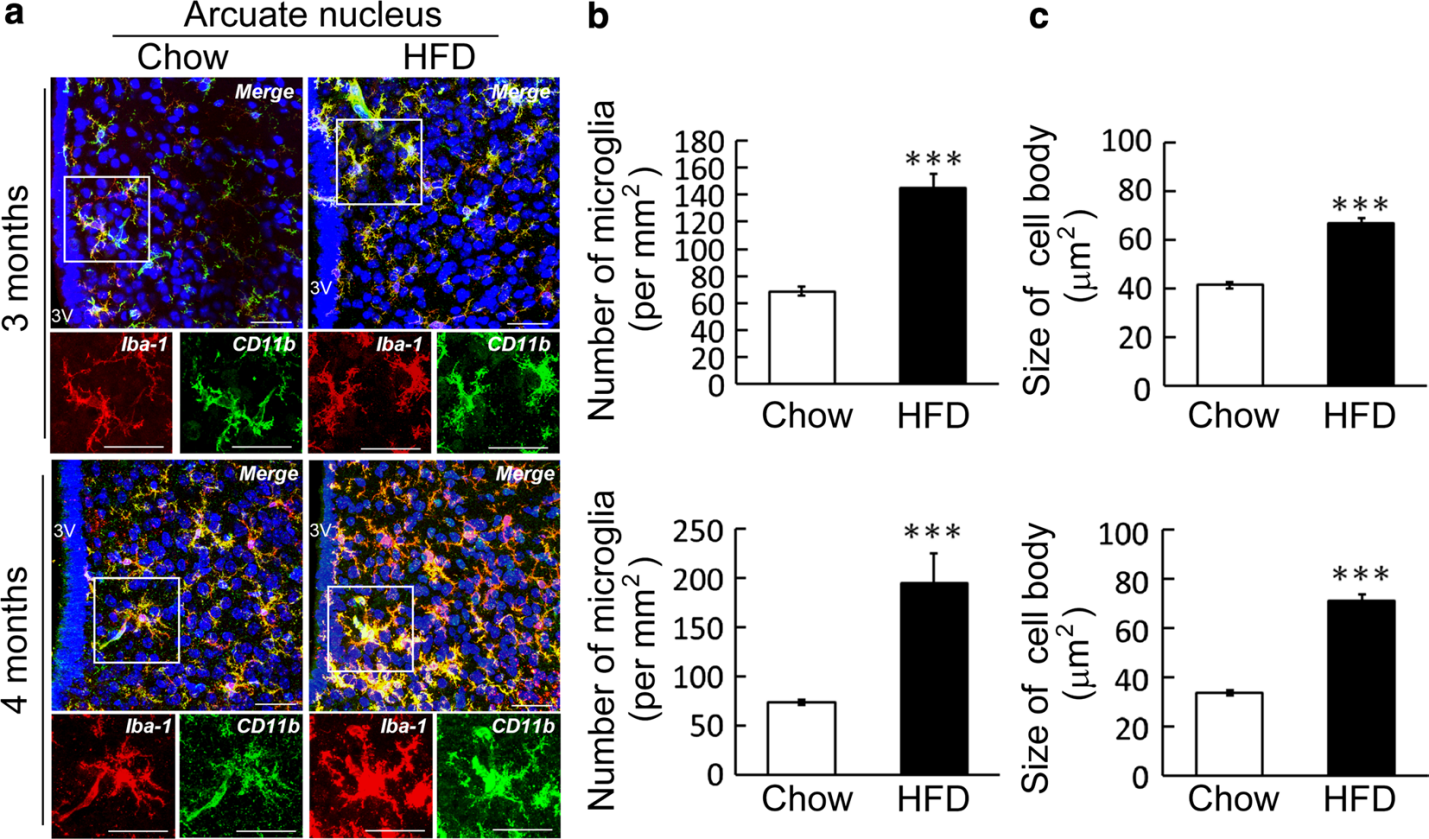
Microglia activation induced by chronic HFD feeding in hypothalamic arcuate nucleus. Mice at the age of 8 week old were fed by normal diet (Chow) and HFD for 3 and 4 months. **a** The images of immunostaining for Iba1 (red) and CD11b (green) in hypothalamic arcuate nucleus (ARC) at 3 and 4 months demonstrate the co-localization of Iba1^+^ cells with CD11b staining. The images with a high resolution are the representatives of Iba1^+^ cells co-localized with CD11b indicated in the insects. The cell numbers of microglia accumulated in ARC were quantified. The average of microglia cell number in ARC per mm^2^ was increased in the HFD group compared to those detected in the Chow group. **c** The averaged cell body size of ARC microglia was measured. Data are presented as mean ± SEM (n = 5 brain sections from 2 animals at each time point). **p < 0.01, ***p < 0.001 versus the Chow group. Scale bar in **a** 50 μm

To understand if hypothalamic inflammation was in accompany with myelin disruption detected in hypothalamus at the same time points, we measured the expression of proinflammatory cytokines in hypothalamus at the distinct time points. However, we found that the upregulation of IL-1β gene expression in hypothalamus was observed at 2 month after HFD feeding, but not at 3 or 4 month (Additional file 1: Fig. S4A). The change in the relative level of TNF-α, IL-6, and IFN-γ was insignificant at the entire experimental time points (data not shown). Therefore, we were next to investigate if microglia accumulation in hypothalamus endured during chronic feeding by HFD. It was evident that Iba1^+^/CD11b^+^-microglia with a hypertrophic shape were intensively located at hypothalamic ARC after HFD feeding for 3 and 4 months (Fig. 2a). Microglia increased in number was also observed at 2 month after HFD feeding (Additional file 1: Fig. S4B). The quantification of Iba1^+^/CD11b^+^-microglia and measurement of Iba1^+^/CD11b^+^-cell body sizes at the distinct feeding time points confirmed that microglia activation in hypothalamic ARC was significantly increased over time by chronic HFD feeding (Fig. 2b, c). We also observed Iba1^+^/CD11b^+^-microglia in hypothalamic median eminence (ME) of the Chow- and HFD-fed groups (Additional file 1: Fig. S4C). Yet, the amount of Iba1^+^/CD11b^+^-microglia in ME after feeding with HFD for 3 and 4 months was similar to that observed in the Chow group. Altogether, our results demonstrate that microgliosis was continuously induced in hypothalamic ARC along with the longer time points of HFD feeding.

### IL-33 upregulation in hypothalamus by chronic HFD feeding

**Fig. 3:**
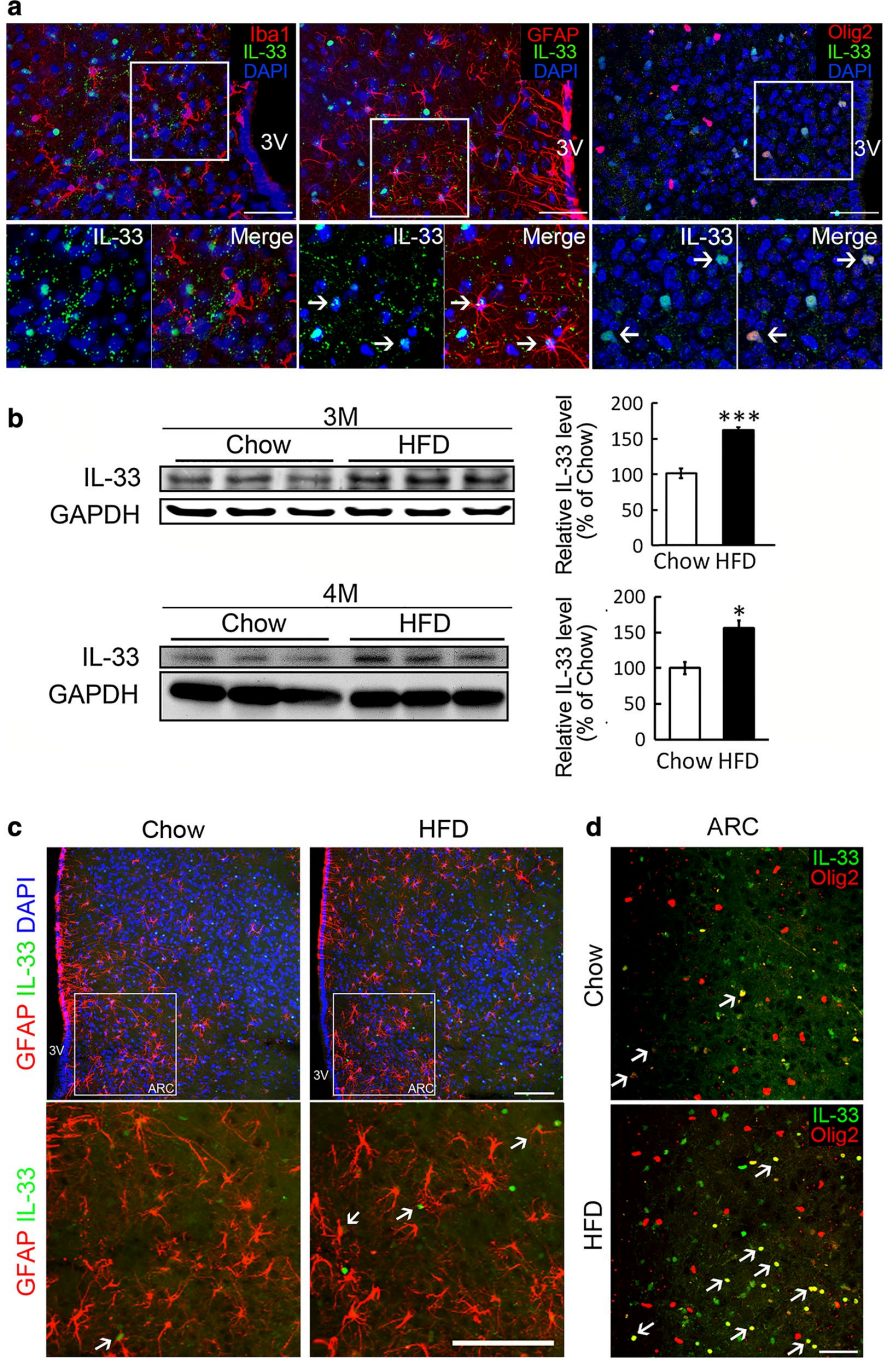
IL-33 in hypothalamus was increased by chronic HFD feeding. **a** The brain sections were prepared from adult mice. The brain sections containing hypothalamic regions were subjected to double immunofluorescence for IL-33 (green) with various glial markers (red) including Iba1 (microglia), GFAP (astrocytes), and Olig2 (OLGs). The nuclear counterstaining by DAPI (blue) was also conducted. The representative images were taken from hypothalamic ARC. The enlarged images from the insets indicated in the upper panel are included in the lower panel. Arrows shown in the lower panel indicate IL-33^+^/GFAP^+^-astrocytes and IL-33^+^/Olig2^+^ OLGs. **b** Total proteins were prepared from hypothalamus of the obese mice at 3 and 4 month after Chow or HFD feeding, and then subjected to western blot analysis (left-hand panel) to examine IL-33 protein expression. IL-33 protein levels were quantified (right-hand panel). **c**, **d** The brain tissue sections were prepared from the Chow- and HFD-fed groups at 4 month after feeding, and then subjected to immunofluorescence. The representative images were captured from ARC regions. Arrows indicate IL-33^+^/GFAP^+^-astrocytes (**c**) or IL-33^+^/Olig2^+^-OLGs (**d**). The results shown in **b** are presented as mean ± SEM (n = 3 animals for each group at each time point). *p < 0.05, ***p < 0.001 versus the Chow group. Scale bar in **a**, **d** 50 μm; in **c** 100 μm

IL-33, one of IL-1 gene superfamily members that plays dual functions in many inflamed diseases, such as cardiovascular diseases, allergy, multiple sclerosis (MS), stroke, and Alzheimer’s disease [20, 21]. IL-33 proteins were mainly detected in the cell nuclei of hypothalamic GFAP^+^-astrocytes (Fig. 3a, arrows) and Olig2^+^-OLGs (Fig. 3a, arrows), but not Iba1^+^-microglia. We further found that feeding with HFD for 3 and 4 months resulted in an upregulation of IL-33 proteins in hypothalamus (Fig. 3b). In parallel, the results from immunofluorescence showed that IL-33^+^-expressing cells increased in hypothalamus were Olig2^+^-OLGs at 4 month after HFD feeding (Fig. 3d, arrows), as well as GFAP^+^-astrocytes (Fig. 3c, arrows). We also noticed that hypothalamic astrocytes displayed a hypertrophic form in response to chronic HFD feedings (Fig. 3c, arrowheads). Given the fact that OLGs and astrocytes release IL-33 as an alarmin in response to CNS damage [22, 23], our results implicate that chronic HFD feeding triggers an increased expression of hypothalamic IL-33 in OLGs and astrocytes, which might be a critical factor to mediate microglia reactivity in hypothalamus.

### Exposure to IL-33 results in the impairment of oligodendrocytic processes

**Fig. 4:**
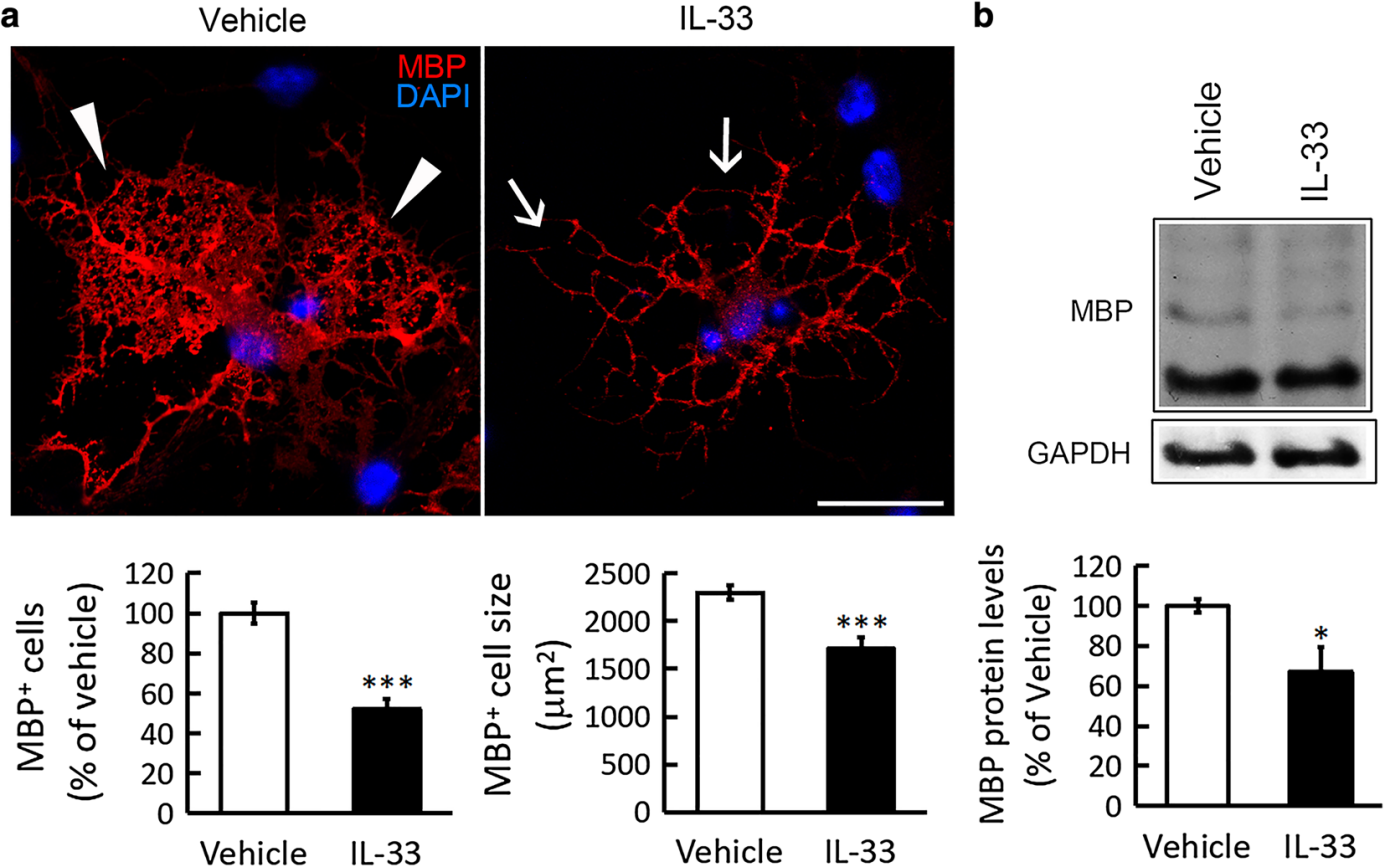
IL-33 induces the morphologic change of oligodendrocytes. **a** Mature OLGs were exposed to 10 ng/ml of IL-33 for 24 h, and then subjected to immunofluorescence (red) using anti-MBP in accompany with DAPI nuclear counterstaining (blue). The interconnected network of OLG processes along with a small cell shape was injured after exposure to IL-33 (arrows). The complex network shape of OLGs in the control culture is indicated by arrowheads. In addition, MBP^+^-OLGs in the cultures treated with vehicle and IL-33 were quantified. The cell size of MBP^+^-OLGs was measured using NIH ImageJ analysis software. **b** Total proteins were prepared from the cultures treated with vehicle and IL-33. The samples were subjected to western blot analysis for the measurement of MBP. Data are presented as mean ± SEM of the three independent experiments. **p < 0.01, ***p < 0.001 versus the vehicle group. Scale bar in **a** 50 μm

IL-33 has been reported to inhibit CNS myelination and regulate the development of MS [18]. Accordingly, our in vivo findings raised the possibility that IL-33 increased in hypothalamus after chronic HFD feeding might mediate the disruption of hypothalamic myelin integrity during chronic HFD feeding. Thus, the in vitro experiments using mature OLGs derived from primary mouse OPCs were conducted to examine if IL-33 induced the morphological change of mature OLGs. As expected, the results showed that exposure to IL-33 for 24 h reduced the complexity of the OLG interconnected web shape (Fig. 4a, arrows). In addition, IL-33 significantly decreased the number of MBP^+^-OLGs in the cultures and diminished MBP^+^-OLG cell size (Fig. 4a). Moreover, MBP protein levels were downregulated in the culture after treatment with IL-33 (Fig. 4b). Similarly, exposure of mature rat OLGs to IL-33 caused significant alteration in the process interconnection of MBP^+^-OLGs (Additional file 1: Fig. S5). The findings reveal that IL-33 is a destructive molecule for the morphological organization of mature OLGs.

## Discussion

Our data show that the integrity of myelin microstructure in the hypothalamic region was damaged after chronic HFD feeding, indicating that myelin injury occurs at the later time point after HFD feeding than microglia activation was observed. Moreover, an increase in IL-33 that is mainly expressed in astrocytes and OLGs was induced in hypothalamus at 3 and 4 month after HFD feeding. The addition of IL-33 to mature OLGs in culture attenuated OLG cell size and MBP expression.

The results that myelin microstructure was impaired by chronic HFD feeding for 4 and 6 months, indicating that myelin injury occurs at much later time phase than the initial time points when an increase in proinflammatory cytokine expression and microglia activation enabled to be detected. This myelin disruption can be prevented when animal food was changed back to normal diet at 3 month after HFD feeding (HFD withdrawal). Although the mechanisms underlying HFD withdrawal-induced maintenance of myelin integrity is not yet defined, our findings provide a fact that HFD feeding can result in the microstructural alteration of myelin in hypothalamus, and HFD feeding for a longer period might be a risk to induce loss of myelin in hypothalamus. In support of our view, loss of myelin-enriched white matter integrity in individuals with high body mass index (BMI) and metabolic syndrome has been observed through the assessment of diffusion tensor imaging [19, 24]. In addition, it has been reported that maternal obesity can cause inflammation and reduce myelination in the brain of the offspring [25]. Moreover, the findings have demonstrated that HFD intake and sedentary habit can cause loss of OLGs in the spinal cord [15]. Nevertheless, since the loss of Olig2^+^-OLGs in hypothalamus of obese mice was not evident, it is interesting to further determine the mechanisms involved in the injury to hypothalamic myelin structure and in metabolic imbalance of hypothalamic OLGs in chronic obesity. In addition, the recovery of neuronal function along with the preservation of myelin integrity by HFD withdrawal needs to be further disclosed.

Evidence has shown in the rodent models that the expression of the pro-inflammatory cytokines (i.e. TNF-α, IL-6, and IL-1β) in hypothalamus was increased by HFD feeding at the acute phase (from 1 day to 1 week), and then declined at the later time points [11, 12, 26]. Indeed, only IL-1β mRNA expression was significantly upregulated in hypothalamus at 2 month, and induced at an increased trend at 3 and 4 month after HFD exposure (Additional file 1: Fig. S4A). It is also evident that microglia were accumulated with an increase in number in hypothalamic ARC at 2 month after HFD feeding (Additional file 1: Fig. S4B). Thus, it seems like that IL-1β might be one of the critical players in hypothalamic inflammation at the earlier time points. However, the regulators to induce microglia accumulation at the later time points (i.e. 3 and 4 month after HFD feeding) remain unknown. We show here that IL-33, a nuclear alarmin of the IL-1 cytokine family, was increased in hypothalamus of obese mice fed by HFD for 3 and 4 months. Moreover, the findings from others have shown that after CNS injury glial-derived IL-33 acts on microglia via ST-2 receptor to induce chemokines for recruitment of peripheral monocytes [22], or release anti-inflammatory cytokine IL-10 [23]. ST-2 is mainly expressed in astrocytes and microglia [27]. Similarly, examination of ST-2 mRNA expression in cultured glial populations using QPCR indicated that ST-2 is highly expressed in microglia and astrocytes, but lesser in mature OLGs maintained in DM for 7 days (data not shown). IL-33 released from astrocytes and OLGs after CNS injury has been speculated to play a protective role through the regulation of microglial activities [23]. Yet, OLGs-derived IL-33 has been reported to mediate neuropathic pain induced by chronic constriction injury of the sciatic nerve [28]. Thus, the possibility that an increase in IL-33 after chronic HFD feeding might serve as a potential factor to mediate microglia reactivity and induce the reduction in the production of proinflammatory mediators in hypothalamus remains to be further studied.

The recent findings have addressed that IL-33 is a critical factor for MS development via inhibiting CNS myelination [18]. Although ST-2 is expressed in mature OLGs at a much lower level than that in microglia and astrocytes, the findings from others show that ST-2 expression was detected in myelinating OLGs in vivo and in vitro [18]. Moreover, the addition of exogenous IL-33 to myelinating co-culture prepared from embryonic spinal cord tissues can cause a decrease in the amount of myelinated axons, suggesting that exogenous IL-33 is likely to be detrimental for myelinogenesis [18]. We show that hypothalamic IL-33 expressing cells are astrocytes and OLGs in the Chow-fed and HFD-fed groups. Thus, an increase in hypothalamic IL-33 expression at 3 and 4 month after HFD feeding shed light on a the regulatory role for astrocytes and OLGs in hypothalamic inflamed microenvironment at the chronic time stage of HFD feeding. The findings from our laboratory indicated that the absence of IL-33 expression in OPCs can suppress the differentiation of OPCs into mature OLGs [29]. Interestingly, the results from our in vitro study using mature OLGs show that exposure of mature OLGs to extracellular IL-33 impaired the morphology of OLGs along reduced expression of MBP. This suggests that IL-33 plays the different biological roles in the functions of OPCs and mature OLGs. Nonetheless, while the actions of nuclear IL-33 on mature OLGs is still unclear, the insult of mature rodent OLGs by exposure to recombinant IL-33 proteins provides important evidence that an elevated production of IL-33 in hypothalamus after chronic HFD feeding might play the regulatory factor in myelin impairment. Thus, it is possible that IL-33 upregulation in hypothalamus during chronic HFD feeding could not only act as an alarmin to modulate obesity-associated hypothalamic inflammation, but plays a detrimental role in the maintenance of myelin structure.

## Conclusions

In summary, it is the first study to demonstrate that hypothalamic myelin disruption and the upregulation of glia-derived IL-33 were associated with HFD-induced obesity. Our future study is to dissect the role of IL-33 in hypothalamic neuropathogenesis associated with chronic HFD feeding.

## Methods

### Animals

All animal experiments were conducted in accordance to ARRIVE Guidelines (Animal Research: Reporting In Vivo Experiments). Animal care and use was approved by the National Cheng Kung University Institutional Animal Care and Use Committee, Tainan, Taiwan (IACUC approval number: 106060). Eight-week old male C57BL/6 mice (19.91 ± 0.29 g) were obtained from National Cheng Kung University Laboratory Animal Center (http://www.ncku.edu.tw/animal/eng/nckulac.html), and placed in the individual cages (a pair per cage) with free access either to normal diet (Laboratory Rodent Diet #5001; LabDiet, St. Louis, MO, USA) or HFD (Rodent Purified Diet #58Y1; TestDiet, St. Louis, MO, USA). The mice were fed by normal diet or HFD (Additional file 1: Table S1) for the distinct time periods (3, 4, and 6 months). The HFD contains 61.6% kcal from fats, 18.1% from proteins and 20.3% from carbohydrates. The animals were housed (2 animal in a cage) under standard room condition (room temperature: 23 ± 2 °C; humidity: 58 ± 2%; 12-h light/dark cycle) with free access to food and water ad libitum. Animals were sacrificed at different time points (Additional file 1: Table S1), by intraperitoneal (i.p.) injection with Zoletil 50 (Virbac Taiwan Co., Ltd.; 5X dilution in saline, 0.05–0.06 ml/10 g).

### Preparation of primary oligodendrocyte precursor cells (OPCs)

Pregnant C57BL/6 mice or Sprague–Dawley (SD) rats were purchased from BioLASCO Taiwan Co. Ltd. Animal use followed by ARRIVES Guidelines and approved by the National Cheng Kung University Institutional Animal Care and Use Committee, Tainan, Taiwan (IACUC approval number: 103060 and 104273). The animals were sacrificed by injection with Zoletil-50 (0.1 ml/100 g for rats; 0.05–0.06 ml/10 g by 5X-diluted Zoletil-50 for mice) right before the removal of embryos. Three pregnant animals were used for three independent cell preparations to complete this study. OPCs were prepared following the method as previously described [30]. OPCs that were generated from neural stem cells derived from the brain tissues of mouse embryos at 14.5 days were plated onto poly-d-lysine (PDL; Sigma; Cat# P0899) -coated dishes and maintained in the growth medium consisting of DMEM/F12 medium (Thermo Fisher, Cat# 12400024), 2% B27 supplement (Thermo Fisher; Cat# 17504044), 1% N2 supplement (Thermo Fisher; Cat# 17502048), 10 ng/ml epidermal growth factor (ProSpec; Cat# GFH-26-100), 10 ng/ml fibroblast growth factor-2 (Cell Guidance Systems; Cat# PPH146), and 10 ng/ml platelet-derived growth factor-AA (ProSpec; Cat# CYT-341). To induce the differentiation of OPCs into mature OLGs with expression of myelin proteins [31], OPCs were cultured for 7 days in OLG differentiation medium (DM) containing DMEM medium (Thermo Fisher; Cat# 12100-046), 1% Penicillin–Streptomycin (Thermo Fisher; Cat#15140-122), 1 mM sodium pyruvate (Sigma; Cat# P2256), 0.1% bovine serum albumin (Sigma; Cat# A7030), 50 μg/ml apotransferrin (Sigma; Cat# T2252), 5 μg/ml insulin (Sigma; Cat# I6634), 30 nM sodium selenite (Sigma; Cat# S5261), 10 nM biotin (Sigma; Cat# B4639), 10 nM hydrocortisone (Sigma; Cat# H0888), 15 nM triiodothyronine (Sigma; Cat# T6397), 10 ng/ml ciliary neurotrophic factor (ProSpec; Cat# CYT-272), and 5 μg/ml N-acetyl-cysteine (Sigma; Cat# A9165). To determine the effect of IL-33 on OLG maturation, OLGs after a 6-day incubation in DM were continuously treated with mouse recombinant IL-33 (CellGS, Cat# GFM36) in DM for 24 h.

### Western blot analysis

The cells were lysed in lysis buffer (1% tritonX-100, 0.05%Tween-20 in PBS) containing a protease inhibitor cocktail (Thermo Scientific, Cat#78443). Hypothalamic tissues were removed from animals and lysed by T-PER tissue protein extraction reagent (Thermo Scientific, Cat# 78510) containing protease inhibitor cocktail. 100 μg of total proteins were loaded onto 10 or 15% SDS-PAGE, and transferred to a nitrocellulose membrane. The nitrocellulose membranes were immunoblotted overnight at 4 °C with primary antibodies (Table 1). The membrane was then incubated with secondary antibodies conjugated with peroxidase for 60 min at room temperature. The immunoreactive proteins were detected using ECL regent and visualized with Super RX-NC films (Fuji, Japan). Western Lightning^®^ Plus-ECL enhanced chemiluminescent substrate was from PerkinElmer Life Sciences (Boston, MA, USA).

**Table 1:** List of antibodies in the study for immunofluorescence or western blot analysis

Antibodies	Manufacturer and RRID	Immunogen	Working dilution
Monoclonal mouse anti-MBP (SMI-99)	Millipore Cat#NE1019, RRID:AB_2140491	Purified human myelin basic protein with amino acids 131–136	1:200 (IF)
Monoclonal mouse anti-MBP (SMI-94)	Millipore Cat#NE1018, RRID:AB_2140494	Purified human myelin basic protein with amino acids 70–89	1:1000 (WB)
Polyclonal rabbit anti-PLP	Abcam Cat#ab28486, RRID:AB_776593	Synthetic peptide as amino acids 109–128 of mouse myelin PLP	1:1000 (WB)
Polyclonal rabbit anti-Iba1	Wako Cat#019-19741, RRID:AB_839504	Purified by the antigen affinity chromatography from rabbit antisera	1:200 (IF)
Monoclonal rat anti-CD11b	BD Biosciences Cat#550282, RRID:AB_393577	Rat (DA) IgG2b, κ in mouse splenic cells	1:200 (IF)
Polyclonal rabbit anti-Olig2	Millipore Cat#AB9610, RRID:AB_570666	Recombinant mouse Olig-2	1:200 (IF)
Polyclonal rabbit anti-GFAP	Millipore Cat#AB5804, RRID:AB_2109645	Purified bovine GFAP	1:200 (IF)
Polyclonal goat anti-IL-33	R and D Systems Cat#AF3626, RRID:AB_884269	*Escherichia coli*-derived recombinant mouse IL-33	1:200 (IF)
Monoclonal mouse anti-IL-33 (Nessy-1)	Enzo Life Sciences Cat#ALX-804-840/1, RRID:AB_11000255	Recombinant human IL-33 (aa 112-270) (Prod. No. ALX-522-098)	1:1000 (WB)
Monoclonal mouse anti-β-actin	Santa Cruz Biotechnology Cat#sc-81178, RRID:AB_2223230	Recombinant protein corresponding to a region near the C-terminus of β-Actin of human origin	1:1000 (WB)
Monoclonal mouse anti-GAPDH	Millipore Cat#MAB374, RRID:AB_2107445	Glyceraldehyde-3-phosphate dehydrogenase from rabbit muscle	1:2000 (WB)

*IF* immunofluorescence, *WB* western blot

### Immunofluorescence

The brain tissues were post-fixed by 4% PFA overnight, and then cryoprotected in 30% (w/v) sucrose in PBS. The tissues were embedded in Tissue Tek optimal cutting temperature compound (Electron Microscopy Sciences, Torrance, CA, USA), sectioned with a 20-μm thickness. The free-floating coronal brain sections were treated with 1% TritonX-100 in PBS at 4 °C overnight, and incubated with the primary antibodies in PBS containing 0.1% TritonX-100 and 1% horse serum at 4 °C overnight. The tissues were subsequently incubated with appropriate biotinylated secondary antibodies for 1 h followed by Alexa488/Cy3–avidin (1:200) for 45 min. For double immunofluorescence, after incubation with the second primary antibody, Alexa488/Alexa594-secondary antibodies (1:200) was added for 1 h. Alternatively, the cells were fixed with 4% PFA for 10 min, and incubated in PBS containing 0.1% Triton-X100 for 30 min. The cultures were incubated overnight at 4 °C with primary antibodies. The immunostained tissues and OLGs were then subjected to DAPI nuclear counterstaining. The staining was observed under an Olympus FLUOVIEW FV1000 confocal laser scanning microscope (Olympus, Japan) with a 405, 488 or 594 nm lasers. The antibodies and biotinylated secondary antibodies used in the study were listed in Table 1.

### TEM imaging

Mice were perfused by 0.9% saline and the fixation solution containing 2% glutaraldehyde and 2% PFA in 0.1 M phosphate buffer. The pieces of hypothalamic tissue at 1 mm^3^ prepared from lateral region adjunct to hypothalamic ARC area (Additional file 1: Fig. S1) were sectioned at the thickness of 100 nm for Hitachi HT-7650 Transmission electron microscopy imaging system (Tokyo, Japan) operated by the core facility of National Health Research Institutes (NHRI, Taiwan).

### Quantification of microglia number and cell body

In addition to the morphological observations of Iba1^+^ or CD11b^+^ microglia in hypothalamus, microglia activation was evaluated by measurement of microglia number and cell body size by NIH ImageJ analysis software. Five randomly selected images per brain section were merged captured in multiple steps of 1-μm thickness using Olympus FLUOVIEW FV1000 confocal laser scanning microscope. Five brain sections containing hypothalamic ARC regions were collected from two animals in each group at each time point. The number of Iba1^+^ microglia and the average cell body size of the Iba1^+^ microglia in the hypothalamic ARC regions (Additional file 1: Fig. S1) were quantified.

### Evaluation of oligodendrocytic morphological change

Five randomly sampled images were captured from each culture using an epifluorescence microscope with a 40X objective lens. In addition, MBP^+^-OLGs in the cultures (1000 cells in total per treatment) were quantified, and the cell size of MBP^+^-OLGs in the culture (100 cells in total per treatment) were measured using NIH ImageJ analysis software (RRID: SCR_003070). The results are presented as the percentage of the data obtained in cytokine-treated culture over the vehicle culture.

### Quantitative real-time polymerase chain reaction (QPCR)

All of the reagents used for this assay were prepared in diethylpyrocarbonate (Sigma; Cat# D5758)-ddH_2_O. The animals were sacrificed and perfused by 0.9% saline. Hypothalamic tissues were removed and subjected to RNA isolation using TRIzol™ (Invitrogen; Cat# 15596018). One μg of RNA was reacted with MMLV reverse transcriptase (Invitrogen; Cat# 28025-013) to generate cDNA. PCR amplification was performed using the specific primers and Fast SYBR^®^ Green Master Mix (Applied Biosystems; Cat# 4385612) for 10 min at 95 °C, followed by 40 cycles set for 10 s at 95 °C, annealing for 10 s at 65 °C, and extending for 2 s at 72 °C. The primers used in this study were designed using the Primer-BLAST software provided by National Center for Biotechnology Information, and synthesized by Genomics (Taipei, Taiwan). The expression level of cyclophilin A (CyPA) was used as an internal control. StepOne Software v2.1 (Applied Biosystems) was used to determine the cycle-threshold fluorescence values. The expression level of the target genes relative to the internal control was presented as 2^−ΔCT^, where ΔCT = (Ct_target gene_ − Ct_CyPA_). The sequences of the specific primers for IL-1β and Cyclophilin A (CyPA) are shown as below. Mouse IL1β (NM_008361.4): forward: 5′-TGCCACCTTTTGACAGTGATGA-3′, reverse: 5′-AAGGTCCACGGGAAAGACAC-3′; mouse CyPA (NM_008907.1): forward: 5′-CGTCTGCTTCGAGCTGTTTG-3′, reverse: 5′-GTAAAATGCCCGCAAGTCAA-3′.

### Statistical analysis

The presence of significant differences between the two groups (Chow and HFD) at single time point observed in this study was determined using two-tailed Student’s *t* test. Each value is the mean ± SEM from five animals per group (MBP and PLP expression), three animals per each time point per animal group (IL-33 expression), five brain sections containing hypothalamic ARC regions (Iba1 and CD11b immunofluorescence), and three repeated experiments using OLG cultures prepared by the three preparations (in vitro study). The statistical significance was set as *p < 0.05; **p < 0.01; ***p < 0.001.

## Additional file

**Additional file 1: Table S1.** A list of animals used for the assays indicated in figures and supplementary figures; **Figure S1.** Anatomical diagram; **Figure S2.** Examination of myelin structure in the hypothalamus of mice receiving Chow and HFD feeding for 3 months; **Figure S3.** Examination of the body weight, water intake, food intake and calorie intake of mice receiving Chow and HFD; **Figure S4.** Microglia activation induced by HFD feeding in hypothalamic arcuate nucleus at 2 month after feeding; **Figure S5.** Exposure to IL-33 hinders the maturation of rat oligodendrocytes.

## Data Availability

The datasets of this study are available from the corresponding author on request.

## Abbreviations

ARC: arcuate nucleus
BBB: blood–brain barrier
CNS: central nervous system
CyPA: cyclophilin A
DAPI: 4′,6-diamidino-2-phenylindole
HFD: high fat diet
IL: interleukin
MBP: myelin basic protein
ME: median eminence
OLG: oligodendrocyte
PBS: phosphate-buffered saline
PFA: paraformaldehyde
PLP: myelin proteolipid protein
POMC: pro-opiomelanocortin
