# Supplementary material for: Chronic exposure to high fat diet triggers myelin disruption and interleukin-33 upregulation in hypothalamus
Source: BMC Neurosci. 2019 Jul 10;20:33. doi: 10.1186/s12868-019-0516-6 (PMC6617565; doi:10.1186/s12868-019-0516-6)
Supplement: Supplementary file 1 — Additional file 1: Table S1. A list of animals used for the assays indicated in figures and supplementary figures; Figure S1. Anatomical diagram; Figure S2. Examination of myelin structure in the hypothalamus of mice receiving Chow and HFD feeding for 3 months; Figure S3. Examination of the body weight, water intake, food intake and calorie intake of mice receiving Chow and HFD; Figure S4. Microglia activation induced by HFD feeding in hypothalamic arcuate nucleus at 2 month after feeding; Figure S5. Exposure to IL-33 hinders the maturation of rat oligodendrocytes. [file 12868_2019_516_MOESM1_ESM.pdf]

Table S1. A list of animals used for the assays indicated in figures and supplementary figures.

Experiments	Time points	Chow	HFD	Pregnant	Figures
Western Blot assay for MBP and PLP	4m	5/45	5/48		Fig. 1
IF for MBP and Olig-2	4m	1/45	1/48		Fig. 1
TEM imaging	3m	1/45	1/48		Fig. S2
	4m	3/45	3/48		Fig. 1
	6m	2/45	2/48<br>HFD withdrawal:2/48		Fig. 1
IF for Iba1	2m	2/45	2/48		Fig. S4
	3m	2/45	2/48		Fig. S4
	4m	2/45	2/48		Fig. 2
IF for co-localization of IL-33 with Olig2, Iba1, GFAP	4m	2/45	1/48		Fig. 3
Western Blot for IL-33 levels	3m	3/45	3/48		Fig. 3
	4m	3/45	3/48		Fig. 3
QPCR for IL1β mRNA levels	2m	3/45	3/48		Fig. S4
	3m	5/45	5/48		Fig. S4
	4m	4/45	5/48		Fig. S4
	6m	3/45	3/48		Fig. S4
Exposure of primary OLGs to IL-33 proteins				3 (mice)<br>3 (SD rats)	Fig.4<br>Fig. S5
# Body weight, water intake, food intake, calorie intake	Weekly for 6 months	6/45<br>(3 for QPCR;<br>2 for TEM)	5/48<br>(3 for QPCR<br>2 for TEM)<br>*HFD withdrawal: 4/48<br>(2 for TEM)		Fig. S3
Total		45	48	6	

1. Male C57BL/6 mice at 8 weeks old were randomly divided into two groups for Chow or HFD feeding.

2. For HFD withdrawal group (*), the mice were randomly taken among animals in HFD-fed group at 3 month after HFD feeding, and then fed by Chow.
3. #: One animal in the Chow group and 2 animals in HFD withdrawal group were used for the technical training of TEM tissue preparation.
4. The sample size decision was following the 3Rs (Replacement, Reduction and Refinement) and minimize the use of animals to avoid unnecessary sacrifice.

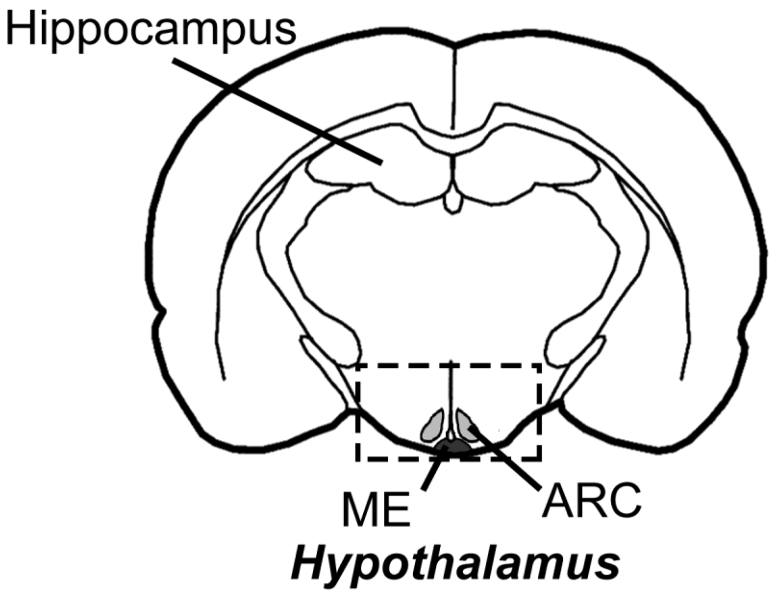

Figure S1. The anatomical diagram indicates the hypothalamic ARC and ME that were studied to measure the amounts of microglia, and shows the areas (dash lines) around hypothalamic ARC for immunofluorescence and TEM imaging analysis.

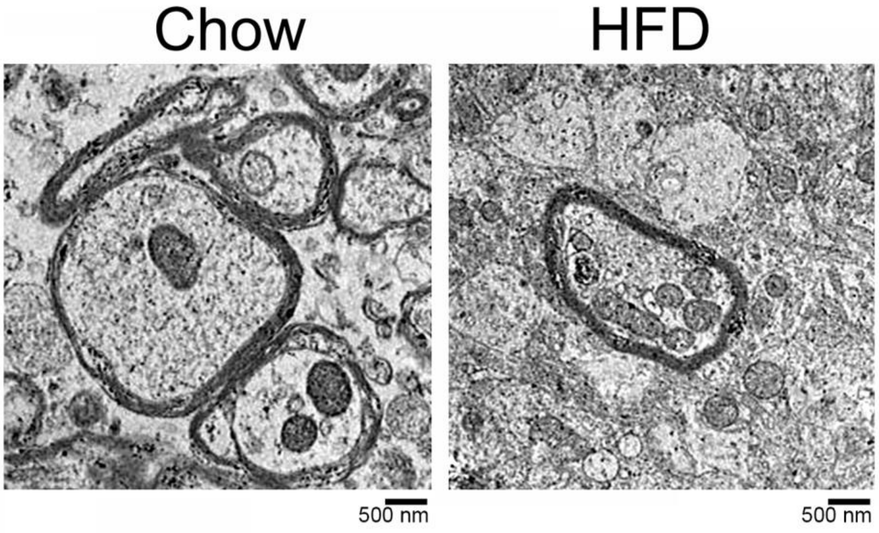

Figure S2. Examination of myelin structure in the hypothalamus of mice receiving Chow and HFD feeding for 3 months. The hypothalamic tissues were prepared from Chow and HFD-fed mice and then subjected to TEM imaging analysis. The compacted structure of myelin was still observed in the hypothalamus from obese mice. Scale bar, 500 nm.

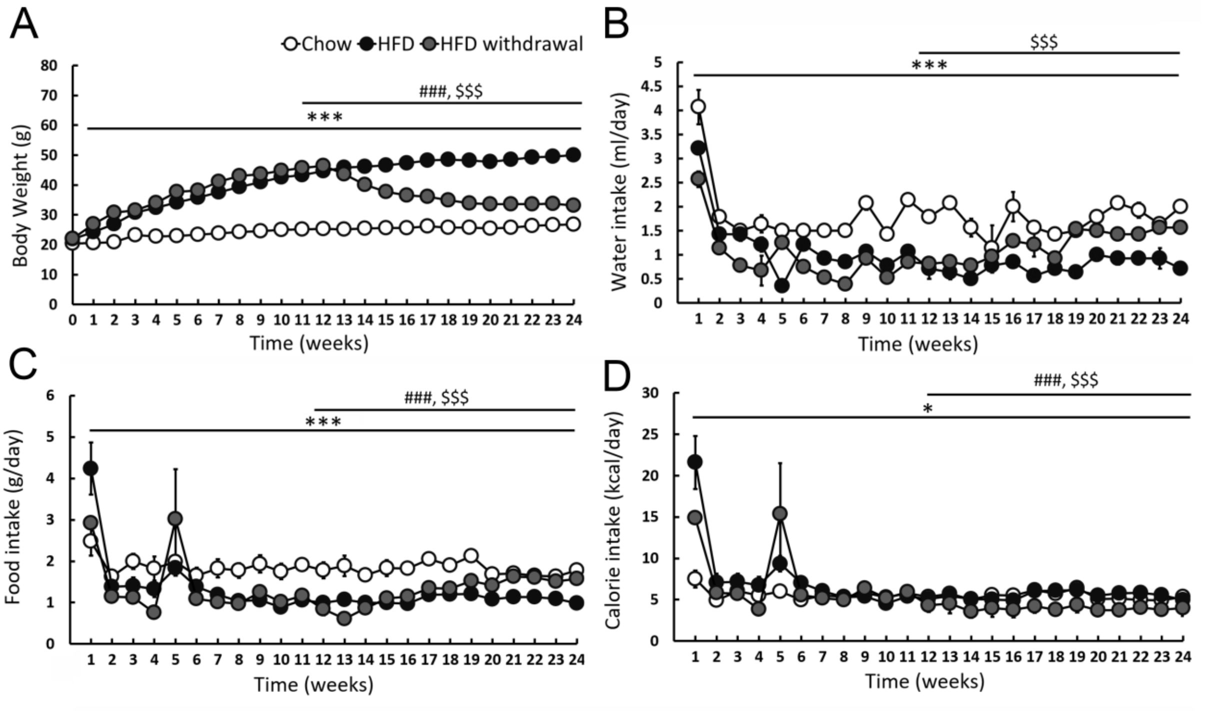

Figure S3. Examination of the body weight, water intake, food intake and calorie intake of mice receiving Chow and HFD. Mice (n = Chow: 6, HFD: 5, HFD withdrawal: 4) were fed by Chow and HFD up to 6 months (24 weeks). Alternatively, mice were fed by HFD for 3 months, and then their diet changed to Chow for another 3 months. The body weight (A), water intake (B), and food intake (C) were measured every week. The data of calorie taken from food was measured as conversion of diet (D). HFD-induced increase in the body weight of mice with reduced water and food intake compared to those analyzed in the Chow group. Note that the change in calorie taken from diets in each group was not significant (n.s.). *p<0.05, ***p<0.001 HFD versus Chow. ###p<0.001 HFD withdrawal versus HFD. $$$$p$<0.001 HFD withdrawal versus Chow.

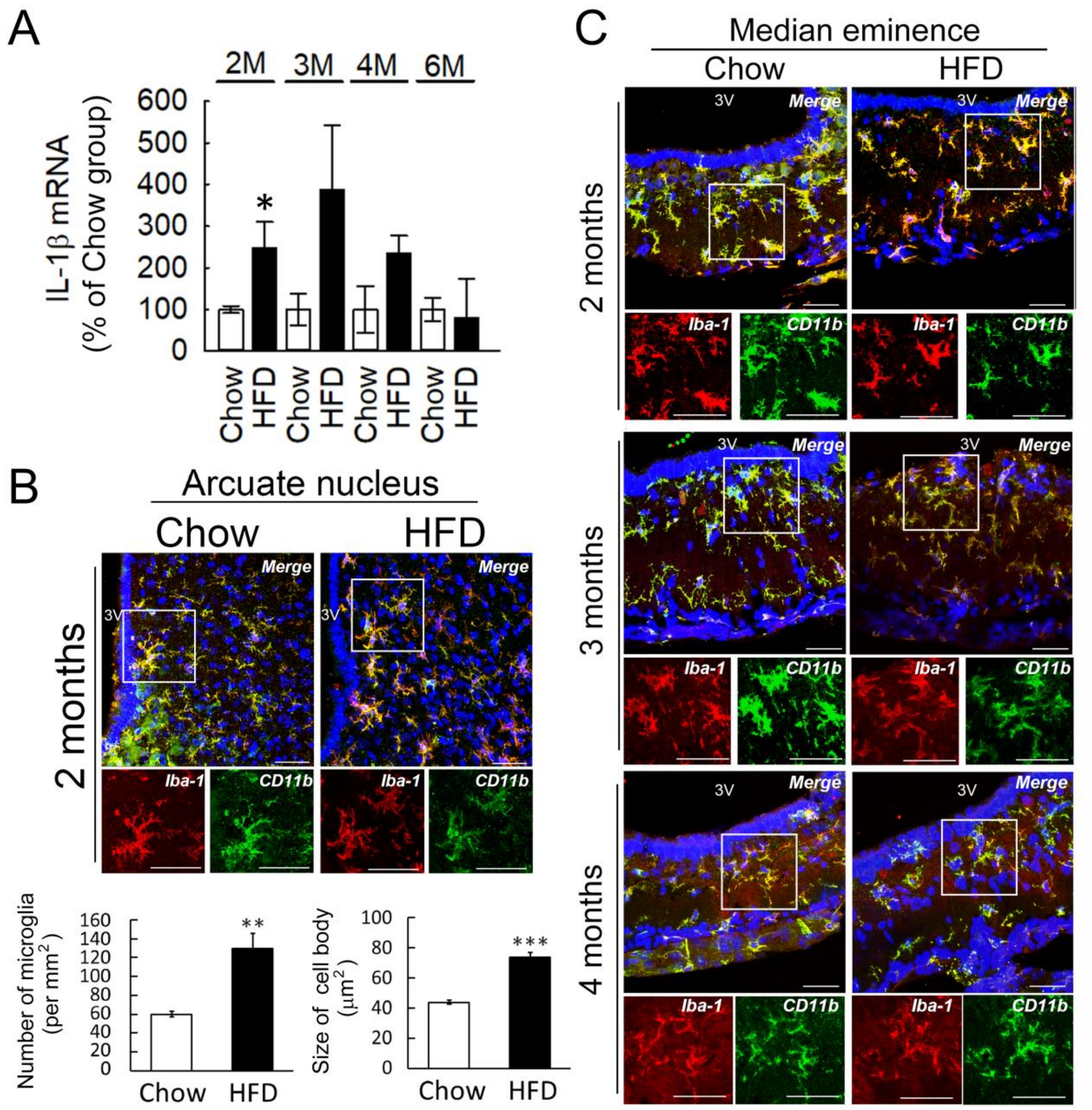

Figure S4. Microglia activation induced by HFD feeding in hypothalamic arcuate nucleus at 2 month after feeding. (A) Mice at the age of 8 week old were fed by normal diet (Chow) and HFD for the indicated time periods. Total RNA was isolated from hypothalamus, and then subjected to QPCR for the measurement of IL-1β mRNA expression. The results indicate that IL-1β mRNA expression was significantly upregulated at 2 month after HFD feeding. Data are presented as mean ± SEM (n = 3 animals for each time point). *p<0.05 versus Chow. (B) Mice at the age of 8 week old were fed by normal diet (Chow) and HFD for 2 months. The brain sections were prepared as described in Materials and Methods, and then subjected to immunofluorescence for Iba1 and CD11b with the nuclear counterstaining by DAPI (blue). The representative images were taken from hypothalamic arcuate nucleus (ARC). The images

with a high resolution are the representatives of $Iba1^+$ cells co-localized with CD11b indicated in the insects. The average of microglia cell number in ARC per mm^2 and the averaged cell body size of ARC microglia were increased by HFD feeding compared to those detected in the Chow group. The results indicate that microgliosis was observed in ARC at 2 month after HFD feeding. Data are presented as mean ± SEM (n = 5 brain sections from 2 animals at each time point). $^{**}p<0.01$, $^{***}p< 0.001$ versus the Chow group. (C) The representative images of immunostaining for Iba1 (red) and CD11b (green) were captured from hypothalamic median eminence (ME) at 2, 3, and 4 month after HFD feeding. We observed no difference in ME microglia cell numbers between Chow and HFD feeding. Scale bar in B and C, 50 μm.

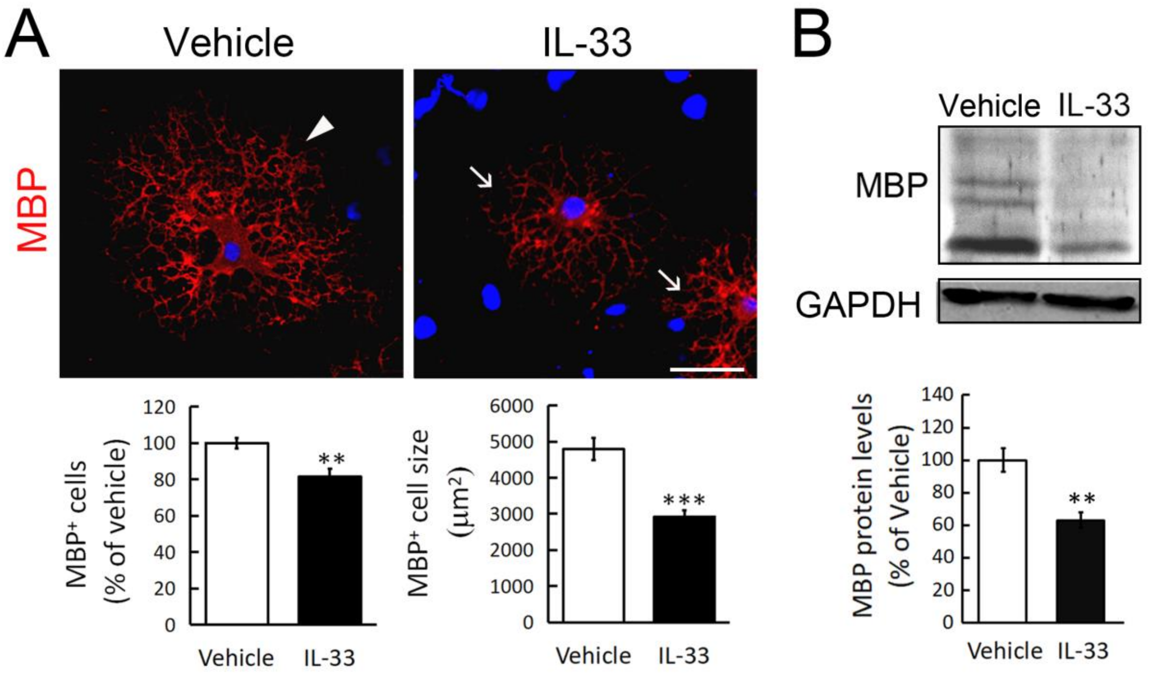

Figure S5. Exposure to IL-33 hinders the maturation of rat oligodendrocytes. (A) Mature OLGs derived from rat OPCs were exposed to 10 ng/ml of recombinant rat IL-33 for 24 h. The cell processes of MBP^+-OLGs were reduced (arrow). The interconnection of OLG cell processes in the control culture was complex (arrowheads). In addition, MBP^+-OLGs were lower in IL-33-treated cultures than that analyzed in the control (vehicle). The cell size of MBP^+-OLGs was also smaller in IL-33-treated cultures than that in the control (vehicle). (B) Total proteins were prepared from the cultures treated with vehicle and IL-33 to measure MBP expression. A decline in MBP levels was detected in IL-33-treated cultures when compared to that measured in the control (vehicle). Data are presented as means $\pm$ SEM of the three independent experiments. ***p*<0.01, ****p*<0.001 versus vehicle. Scale bar in A, 50 μm.
